# Louisville Summer School of Medicine

**Published:** 1845-04

**Authors:** 


### LOUISVILLE SUMMER SCHOOL OF MEDICINE.

The course commenced in this school on the last Monday in March to an increased class. There is manifestly a growing disposition on the part of medical students to remain in the city during the summer, and large classes will reward the efforts of the gentlemen connected with the Summer School if they persevere in the enterprise for a few years. It may be permitted us to say, that the junior members of the association are reaping a valuable reward in the reputation which they are acquiring from their labors. They are acquitting themselves most creditably, and affording in their lectures proof of talents which will make them distinguished teachers.

